# The protective role of MC1R in chromosome stability and centromeric integrity in melanocytes

**DOI:** 10.1038/s41420-021-00499-9

**Published:** 2021-05-18

**Authors:** Xin Li, Weiwei Mao, Jie Chen, Colin R. Goding, Rutao Cui, Zhi-Xiang Xu, Xiao Miao

**Affiliations:** 1 Department of Dermatology, Yueyang Hospital of Integrated Traditional Chinese and Western Medicine, Shanghai University of Traditional Chinese Medicine, 200437 Shanghai, China; 2 Institute of Dermatology, Shanghai Academy of Traditional Chinese Medicine, 201203 Shanghai, China; 3 Department of Dermatology, Longhua Hospital, Shanghai University of Traditional Chinese Medicine, 200032 Shanghai, China; 4 Ludwig Institute for Cancer Research, University of Oxford, Headington, Oxford, OX3 7DQ UK; 5 Department of Dermatology, Second Affiliated Hospital, Zhejiang University School of Medicine, 310009 Hangzhou, China; 6 School of Life Sciences, Henan University, Kaifeng, China; 7 Innovation Research Institute of Traditional Chinese Medicine, Shanghai University of Traditional Chinese Medicine, 1200 Cailun Road, 201203 Shanghai, China

**Keywords:** Mechanisms of disease, Melanoma

## Abstract

Variants in the melanocortin-1 receptor (*MC1R*) gene, encoding a trimeric G-protein-coupled receptor and activated by α-melanocyte-stimulating hormone (α-MSH), are frequently associated with red or blonde hair, fair skin, freckling, and skin sensitivity to ultraviolet (UV) light. Several red hair color variants of *MC1R* are also associated with increased melanoma risk. *MC1R* variants affect melanoma risk independent of phenotype. Here, we demonstrated that MC1R is a critical factor in chromosome stability and centromere integrity in melanocytes. α-MSH/MC1R stimulation prevents melanocytes from UV radiation-induced damage of chromosome stability and centromere integrity. Mechanistic studies indicated that α-MSH/MC1R-controlled chromosome stability and centromeric integrity are mediated by microphthalmia-associated transcription factor (Mitf), a transcript factor needed for the α-MSH/MC1R signaling and a regulator in melanocyte development, viability, and pigment production. Mitf directly interacts with centromere proteins A in melanocytes. Given the connection among *MC1R* variants, red hair/fair skin phenotype, and melanoma development, these studies will help answer a question with clinical relevance “why red-haired individuals are so prone to developing melanoma”, and will lead to the identification of novel preventive and therapeutic strategies for melanomas, especially those with redheads.

## Introduction

Skin color is determined by epidermal melanin, the function of which remains poorly understood. Clinically, there is a lower incidence of melanoma in individuals with high levels of constitutive brown/black pigment and/or acquired pigmentation (e.g., tanning). Conversely, individuals with red hair, blue eyes, and inability to tan are at higher risk for developing melanoma. In the United States, white Americans are 25 times more likely to develop melanoma than African Americans. In Caucasians, melanoma risk is up to tripled in red/red-blonde-haired individuals, compared to dark-haired people. A long-standing clinical question is why red-haired individuals are so prone to developing melanoma.

The melanocortin-1 receptor (MC1R) plays a crucial role in tanning and pigmentation in humans and mice. MC1R is a trimeric G-protein-coupled receptor that is activated by the α-melanocyte-stimulating hormone (α-MSH)^1^. Upon α-MSH binding, MC1R activates the cAMP signaling pathway and promotes melanin production in melanocytes. α-MSH/MC1R signaling also functions in DNA repair after ultraviolet (UV) irradiation^2–4^.

Molecular and genetic data have shown that red hair color variants (RHC-variants) of *MC1R* occur in the coding region of this gene, and are associated with phenotypes, such as red or blonde hair, fair skin, freckling, and skin sensitivity to UV light in humans^5^^,^^6^. In addition, some of these RHC-variants, particularly V60L, I40T, R142H, R151C, R162P, R160W, and D294H, cannot stimulate cAMP production as strongly as wild type and other variants of MC1R in response to α-MSH stimulation.

α-MSH/MC1R is crucial in UV-induced DNA damage repair in melanocytes^7–9^. A change of chromosome number is termed aneuploidy, which is critical for sporadic carcinogenesis and collaborates with intragenic mutations. The centromere is a unique and functional chromosomal domain responsible for the accurate segregation of chromosomes during mitosis^10–13^. Centromere provides a platform or a foundation for the assembly of the kinetochore. Centromeres consist of α-satellite DNA and sequence-specific DNA-binding proteins^12^. There are a number of known centromere-specific binding proteins, which mainly include centromere proteins A (CENP-A), CENP-B, and CENP-C^11^. CENP-A is a self-templating, histone H3 variant, and an integral component of the inner kinetochore that forms a functional centromere^14^. CENP-A functions by directly or indirectly recruiting the major kinetochore nucleating protein CENP-C to maintain centromere position during mitosis through two distinct yet redundant mechanisms^15,16^. The carboxyl-terminus of CENP-A directly recruits CENP-C to centromeres, whereas the amino terminus interacts with CENP-C indirectly through the mediation of CENP-B^14,17^, a centromeric DNA sequence-specific binding protein. CENP-B binds to 17 base pair sequences (called CENP-B boxes) that are interspersed throughout all human centromeres except for the Y chromosome centromere^11,12,18^. Abnormal spindle leads to DNA breaks in the centromere. Centromere-localized breaks usually signal the generation of DNA damage via the mitotic spindle^19^. Centromere breakage may destroy the foundation for the assembly of the kinetochore, leading to improper sister-chromatid segregation and chromosomal instability (CIN)^11,13,20^.

We previously reported that activating MC1R protein palmitoylation is a potential intervention strategy to rescue loss-of-function MC1R in MC1R RHC-variants for therapeutic benefit in vitro and in vivo^21^. In the current study, we demonstrated that α-MSH/MC1R protects melanocytes from accumulating UV-induced chromosome aberrations with a specifically high level of centromeric fragmentations. α-MSH/MC1R-protected chromosome stability and centromere integrity are palmitoylation dependent in melanocytes. Thus, exogenously activated palmitoylation of MC1R RHC-variants protects centromere integrity after UV radiation (UVR) in melanocytes.

## Results

### α-MSH/MC1R functions on centromere integrity after UVR

**Fig. 1:**
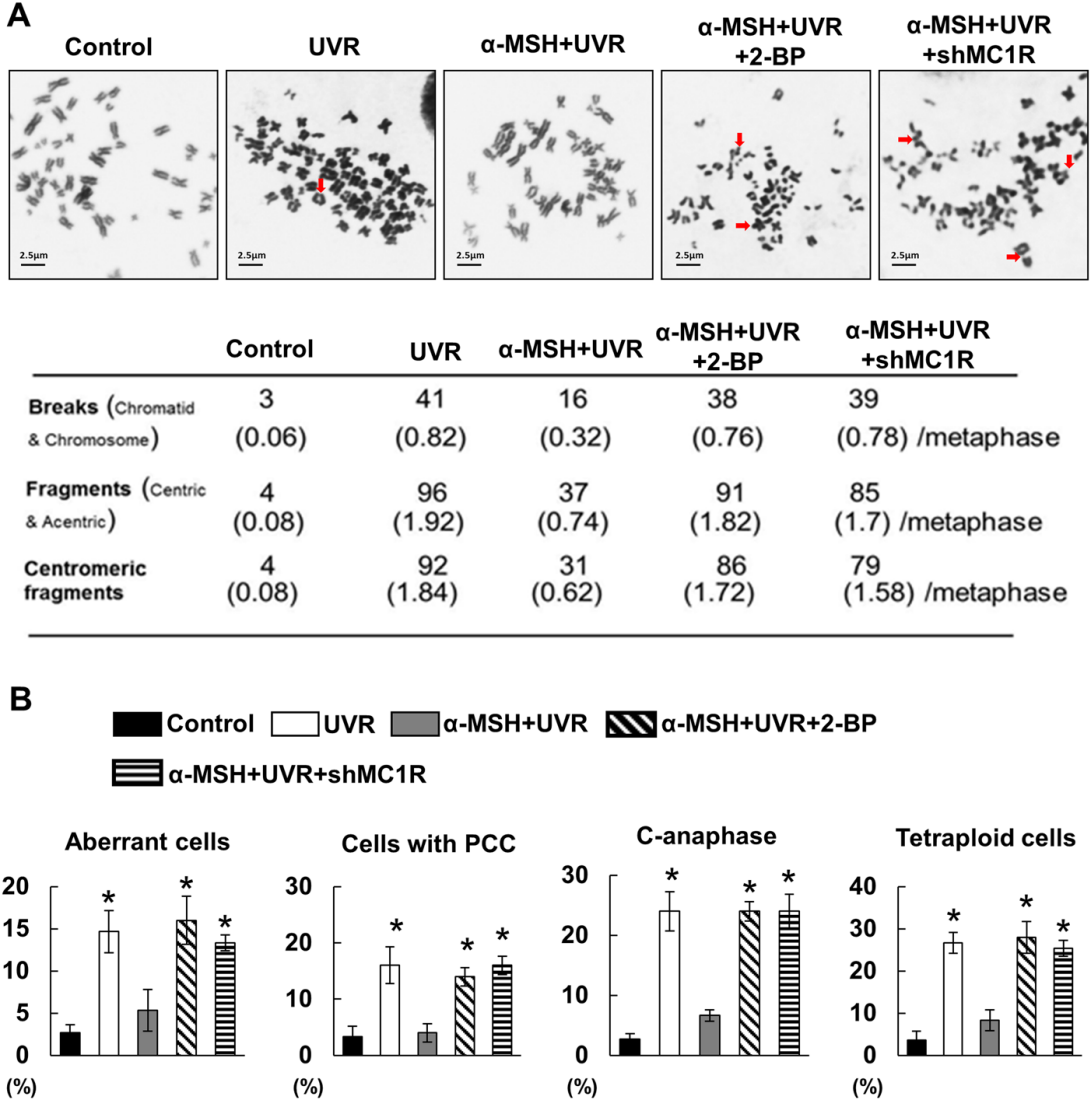
The role of α-MSH/MC1R in the prevention of UVR-induced chromosomal instability and centromere fragmentations in HPMs. **A** Chromosome was analyzed in HPMs stimulated with or without MSH (*n* = 84). Low panel: centromeric fragments were counted as indicated. **B** Multiple cytogenetic abnormalities as indicated were counted in metaphase spread in HPMs.

MC1R plays a role in UVR-induced melanocytic responses, including DNA repair^7–9^. However, it remains unclear whether the level of MC1R impacts genome stability, which is sensitively responded to UVR in melanocytes. We therefore measured chromosome stability and centromeric integrity in MC1R intact, and compromised cells with or without UVB. Human primary melanocytes with either wild-type *MC1R* or *MC1R* silencing were stimulated with α-MSH (10 µM) before irradiation with 100 J/m^2^ UVB, a dosage that generates standard erythema in UVB. Giemsa staining and metaphase spread chromosome analysis were performed in treated cells. Strong cytogenetic alterations were detected in human primary melanocytes after UVR, especially in HPMs with MC1R silencing. Using telomere fluorescence in situ hybridization (FISH) and centromeric FISH, we further validated the chromosome instability in *MC1R*-silenced cells and revealed that centromeric fragmentations were major chromosome aberrances in the cells (Fig. 1). These results indicate a protective role of α-MSH/MC1R in chromosome stability and centromeric integrity after UV irradiation in melanocytes.

MC1R protein palmitoylation is essential for activating MC1R signaling^21^. Thus, we determined whether MC1R signaling-regulated chromosome stability and centromeric integrity are MC1R protein palmitoylation dependent. Chromosome stability and centromeric integrity were detected in melanocytes with or without UVB exposure and/or palmitoylation inhibition. Specifically, human primary melanocytes were stimulated by α-MSH (10 µM) and 2-bromopalmitic acid (2-BrP, 50 µM), a general palmitoylation inhibitor^21^, before irradiation with 100 J/m^2^ UVB. Giemsa staining and metaphase spread chromosome analysis were performed in treated cells. Strong cytogenetic alterations were detected in human primary melanocytes after the pan-palmitoylation inhibition. The protective effect of α-MSH/MC1R on chromosome stability was abrogated when the palmitoylation was inhibited with 2-BrP (Fig. 1). These results indicate that the protective role of MC1R in the chromosome stability and centromeric integrity is MC1R protein palmitoylation dependent.

**Fig. 2:**
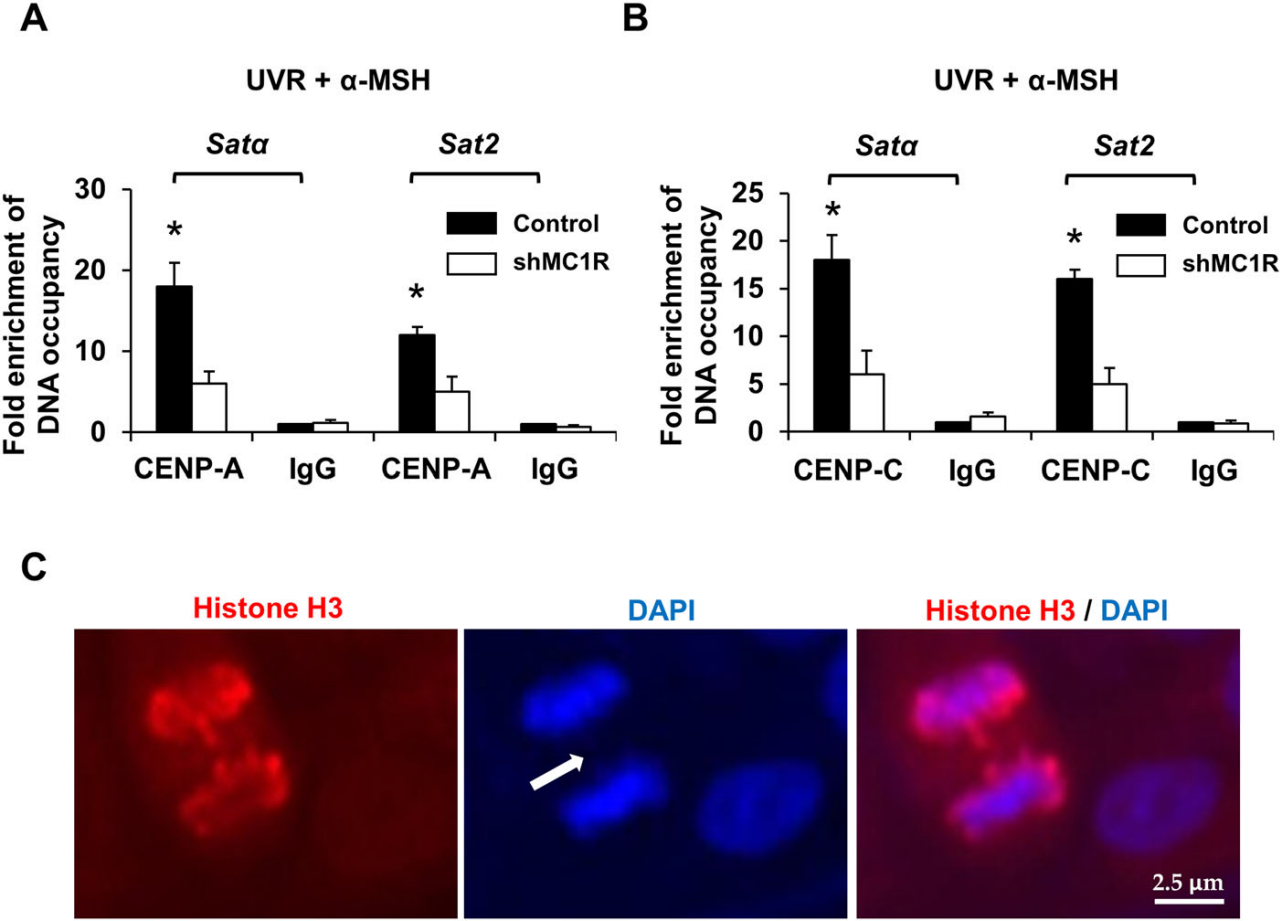
Lack of MC1R disrupts CENP-A/C binding to centromeric and pericentric DNAs. ChIP assays were performed to determine the binding of CENP-A (**A**) and CENP-C (**B**) to centromeric (*Satα*) and pericentric (*Sat2*) DNAs in HPMs/shControl or HPMs/shMC1R, with the Ideal ChIP-seq kit (Diagenode). Anti-CENP-A, anti-CENP-C, or control IgG was used for each IP. After completion of the ChIP, samples were diluted 1:100 in ddH_2_O for qPCR. *Satα* and *Sat2* DNAs were amplified. The value in IgG group was set as “1”. *p < 0.01, *n* = 3, Student’s *t* test. **C** An anaphase HPMs with shMC1R showing lagging chromosome formation (arrow). The nuclei were stained with DAPI (blue).

Centromeric integrity plays a critical role in accurate chromosomal segregation^13,22^. To further identify the role of MSH/MC1R in centromeric integrity, we detected the role of MC1R in the binding of CENP-A/C complex to centromeric and pericentric DNAs. Human primary melanocytes with either wild-type *MC1R* or *MC1R* silencing were stimulated with α-MSH (10 µM) before irradiation with 100 J/m^2^ UVB. Chromatin immunoprecipitation (ChIP) assays were performed to determine the binding of CENP-A or CENP-C to centromeric (*Satα*) and pericentric (*Sat2*) DNAs in HPMs/shR-Ctrl and HPMs/shMC1R, with the Ideal ChIP-seq kit (Diagenode). Specific anti-CENP-A, anti-CENP-C, or control IgG was used for each IP. After completion of the ChIPs, samples were diluted 1:100 in ddH_2_O for qPCR. *Satα* and *Sat2* DNAs were amplified. Lack of MC1R disrupted CENP-A/C binding to *Sata* and *Sat2* DNAs (Fig. 2A, B). In addition, we also observed lagging chromosomes and anaphase bridges during anaphase in division cells in UVR-treated HPMs, in particular in cells with MC1R depletion (Fig. 2C and data not shown). These results further confirm that MC1R is associated with centromere stability and its function. Taken together, our data suggest a protective role of α-MSH/MC1R in chromosome stability and centromeric integrity after UV irradiation in melanocytes, which is palmitoylation dependent.

### Mitf is required in the α-MSH/MC1R-controlled chromosome stability and centromeric integrity after UVR in vitro

**Fig. 3:**
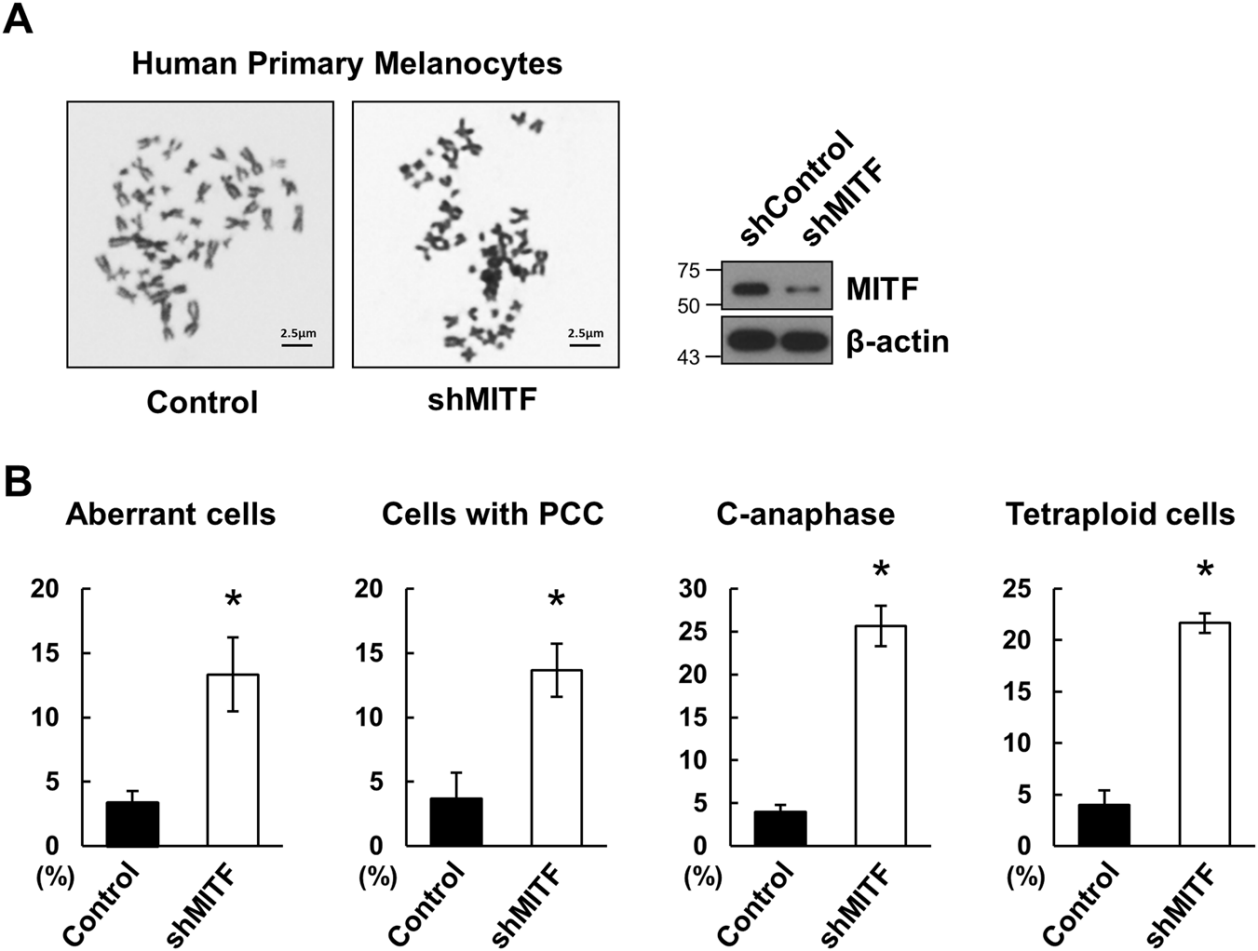
Mitf functions in chromosome stability after UVR. **A** Metaphase spread was performed in HPMs with or without Mitf silencing as indicated. Representative images are displayed. **B** Graphs illustrating the percentage of cytogenetic abnormalities in metaphase spread in **A** from three independent experiments. *n* = 50, error bars represent SEM.

To identify the biochemical mechanism underlying the protective role of α-MSH/MC1R in chromosome stability and centromeric integrity after UVR, we focused on microphthalmia-associated transcription factor (Mitf), a key factor in the α-MSH/MC1R signaling and participated in melanocyte development, viability, and pigment production^23,24^. We first determined the role of Mitf in the protection of chromosomal stability in melanocytes. A Giemsa staining and a metaphase spread chromosome analysis were performed in human primary melanocytes with inducible Mitf silencing. We found that Mitf depletion induced a marked CIN (Fig. 3A).

**Fig. 4:**
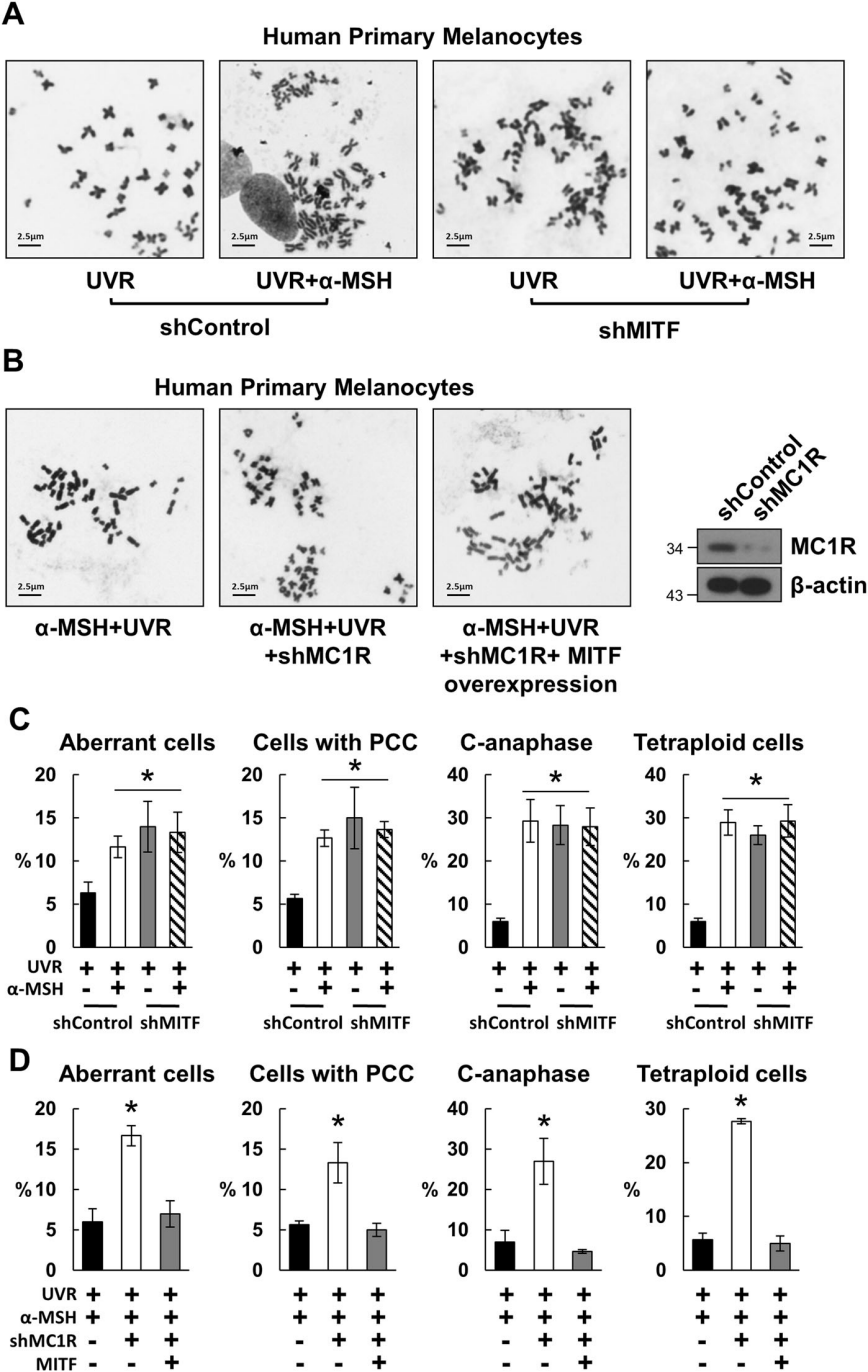
Mitf is required in α**-**MSH/MC1R-controlled chromosome stability after UVR. **A**, **B** Metaphase spread was performed in HPMs with Mitf knockdown (**A**) or Mitf overexpression (**B**). The cells were then irradiated with 100 J/m^2^ UVB. Representative images are displayed. **C**, **D** Graphs illustrating the percentage of cytogenetic abnormalities in **A** and **B** from three independent experiments. *n* = 50, Error bars represent SEM.

To define whether Mitf is required in the α-MSH/MC1R-controlled chromosome stability in melanocytes, a Giemsa staining and a metaphase spread chromosome analysis were performed in human primary melanocytes with stable MC1R silencing and/or Mitf manipulations. We confirmed that MC1R silencing augmented UVR-induced chromosome instability. We also found that CIN was still detected in HPMs with Mitf silencing after α-MSH stimulation (Fig. 4A). On the other hand, Mitf overexpression rescued UVR-induced cytogenetic alterations in HPMs with MC1R silencing (Fig. 4B, C). All these results suggest that Mitf is required in α-MSH/MC1R-controlled chromosome stability in melanocytes.

### Mitf interacts with CENP-A directly in melanocytes

**Fig. 5:**
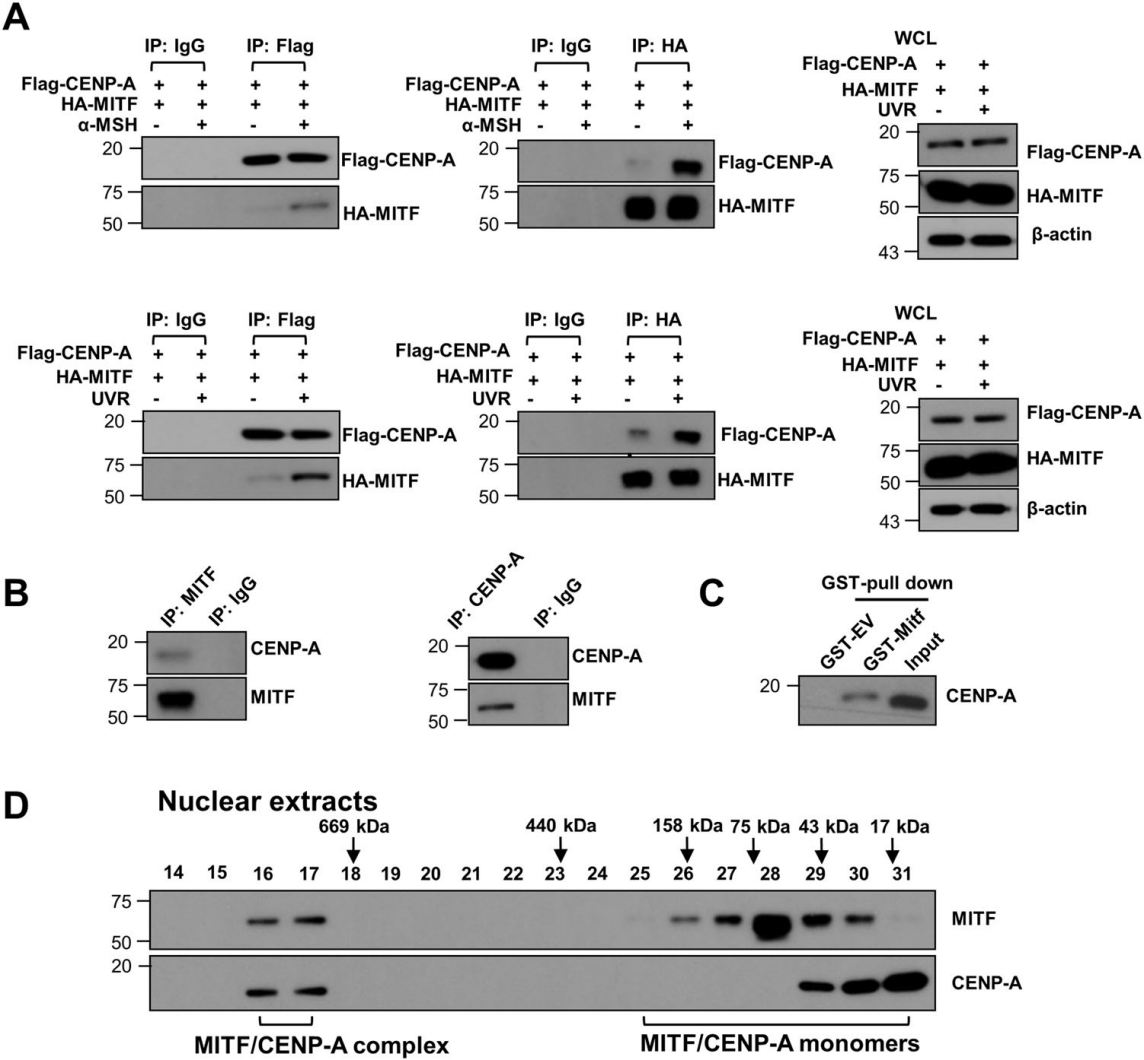
Mitf directly interacts with CENP-A in melanocytes. **A** HA-Mitf and Flag-CENP-A were transduced into HPMs. Binding of Mitf to CEPN-A was confirmed with Co-IP. **B** Interaction between endogenous Mitf and CEPN-A in HPMS was detected with Co-IP using the indicated antibodies. **C** GST-Mitf pulled down CENP-A expressed in HEK cells. Blot was probed with antibody against CENP-A. **D** Whole-cell lysates from HPMs were subjected to the Superdex 200 size-exclusion column to separate proteins at different sizes. The lysates collected at various fractions were separated by SDS–PAGE before immunoblot (IB) analysis.

To further identify how Mitf involves in UVR-induced chromosome stability, we analyzed the potential interaction between Mitf with CENP-A, which contains a histone H3-related histone fold domain that is required for its localization to the centromere. Co-IP was performed using lysates extracted from UVR-exposed melanocytes. We found that endogenous CENP-A pulled down endogenous Mitf in HPMs (Fig. 5A). Administration of α-MSH or exposure to UVB-enhanced Mitf–CENP-A interaction in melanocytes (Fig. 5B). The direct physical interaction between Mitf and CENP-A was further supported by in vitro GST-pull down assay using purified GST-WT-Mitf and recombinant CENP-A protein (Fig. 5C) and by gel filtration assay (Fig. 5D). Taken together, our data suggest that Mitf might mediate α-MSH/MC1R-controlled centromere integrity by interacting with CENP-A in melanocytes.

## Discussion

MC1R is a critical factor in chromosome stability and centromere integrity in melanocytes (Figs. 1 and 2). α-MSH/MC1R stimulation protects melanocytes from UVR-induced damage in chromosome stability and centromere integrity (Fig. 1). The centromere is a specialized domain on the chromosome, appearing during cell division as the constricted central region, where the two chromatids are held together to form an X shape. Thus, the centromere provides a platform or a foundation for the assembly of the kinetochore^25^ and is responsible for the accurate segregation of chromosomes during mitosis. The centromeres consist of α-satellite DNA and sequence-specific DNA-binding proteins. Centromere function is highly conserved and is essential in keeping the integrity of genome. Centromere breakage results in the overexpression of satellite DNA and destroys the foundation for the assembly of the kinetochore, leading to improper sister-chromatid segregation, CIN, and carcinogenesis^25^.

Proteins often work as components of larger complexes to perform a specific function. Characterizing protein complexes can offer important insights into their functions. In the current study, we demonstrated that Mitf, a key factor in α-MSH/MC1R signaling (Fig. 3)^26^, is necessary for chromosome stability and centromere integrity in melanocytes. Interaction between Mitf and CENP-A may play a pivotal role in mediating the role of α-MSH/MC1R signaling in melanocytes (Figs. 4 and 5).

**Fig. 6:**
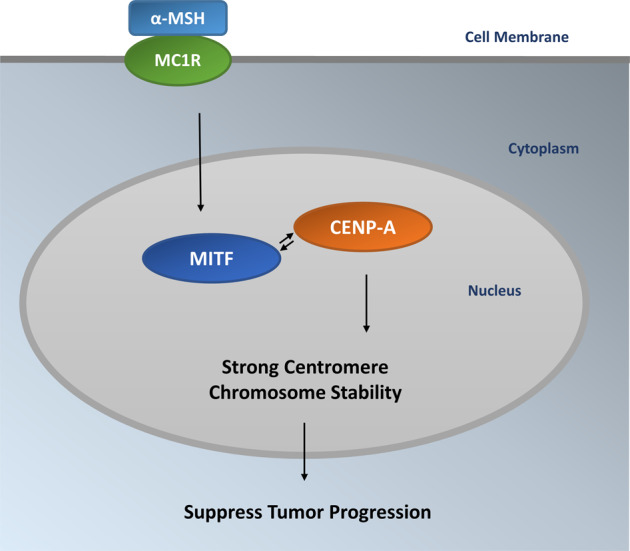
MC1R is a protective factor in chromosome stability and centromere integrity in melanocytes. In human melanocytes, α-MSH binds MC1R and activates MC1R signaling, thus the expression of MC1R downstream factor MITF is stimulated. As a result, the enhanced interaction between MITF and CENP-A protects chromosome stability and reduces the risk of tumor initiation.

*MC1R* gene encodes a trimeric G-protein-coupled receptor activated by α-MSH. Variants in *MC1R* are frequently associated with red or blonde hair, fair skin, freckling, and skin sensitivity to UVR. Several RHC-variants of *MC1R* also associate with increased melanoma risk. Some variants affect melanoma risk independent of phenotype. We previously reported that MC1R protein is palmitoylated and the modification is involved in the maintenance of the activity of MC1R. Activation of MC1R palmitoylation is a potential intervention strategy to rescue loss-of-function MC1R in MC1R RHC-variants for therapeutic benefit in vitro and in vivo^21^. In the current study, we demonstrated that α-MSH/MC1R protects melanocytes from accumulating UV-induced chromosome aberrations with a specifically high level of centromeric fragmentations. α-MSH/MC1R-protected chromosome stability and centromere integrity are palmitoylation dependent in melanocytes. Thus, exogenously activated palmitoylation of *MC1R* RHC-variants may protect centromere integrity after UVR in melanocytes. Given the connection among *MC1R* variants, red hair/fair skin phenotype and melanoma development, our studies will help answer a question with clinical relevance “why red-haired individuals are so prone to developing melanoma” and will lead to the identification of novel preventive and therapeutic strategies for melanomas, especially those with redheads (Fig. 6).

## Materials and methods

### Cell lines, animals, and UV exposure

Cell lines and UV exposure were described previously^27,28^. Primary melanocytes were isolated from normal discarded foreskins, as described previously^21^. Human primary melanocytes were cultured in Medium 254 (Cascade Biology). Adherent cells in a small volume of PBS were irradiated at a dose of 100 J/m^2^ by UVB. After irradiation, PBS was aspirated from the wells, and the cells were fed with media for incubation until the time of the assay^27,28^.

### Plasmids and shRNA constructs

pcDNA3-Flag-MC1R WT and RHC-variants R151C, R160W, and D294H were generated, as previously described^22^. The generation of MC1R expression plasmids and GST-MC1R fusion protein was described previously^29^. shRNA constructs targeting human MC1R (Cat. No. RHS4533-EG4157) or mouse MC1R (Cat. No. RMM4534-EG17199) were purchased from Open Biosystems. The most efficient knockdown cell lines with shmMC1R-A (target sequence: 5′- AATGGAGATCAGGAAGGGATG-3′) or shMC1R-1 (target sequence: 5′-AAATGTCTCTTTAGGAGCCTG-3′) were used for the study.

### Giemsa staining

Dilute Giemsa stain 1:20 with deionbized water. Place slides in May-Grünwald stain (5 min), Trizma® uffer (20–70 mmol/l, pH 7.2, 1.5 min), and then in the diluted Giemsa solution for 15–20 min. The slides were washed in the deionized water and then air dry for evaluation.

### Metaphase spread chromosome analysis

HPMs were passaged into one well of gelatinized six-well dish ~3 days prior to preparation of chromosome spreads. HPMs were treated with Colcemid (1 µg/ml) at 37 °C for 1 h. Cells were treated with 5 ml of ice cold 0.56% KCl solution for 6 min and then fixed with 5 ml of methanol:glacial acetic acid (3:1) fixative solution. Take a small quantity of cell suspension (~20 µl) in a 20 µl pipetter. Release one to three drops in a row onto an alcohol cleaned slide, a single drop at a time. Air dry thoroughly for minimum of 1 h. Prepare at least two slides for each cell line. Photographing and counting chromosomes.

### Chromatin immunoprecipitation

CHIP of the human *Satα* and *Sat2* DNA sequences from HPMs was performed, as described elsewhere^30^. PCR was carried out using primers specific to *Satα* 5′AAGGTCAATGGCAGAAAAGAA and 5′CA ACGAAGGCCACAAGATGTC. *Sat2* primers were purchased from Cell Signaling Technology (Cat. #5077 S).

### Co-immunoprecipitation

Co-IP of Mitf and CENP-A was performed, as described previously^25^. D5 anti-Mitf antibody and EP800Y anti-CENP-A antibody were used for Co-IPs.

### GST-pull down assay

GST-Mitf protein was expressed and purified as described^31^ with minor modification. Briefly, Mitf was cloned into pGEX-6P-1 vector, and then transformed into BL21(DE3) pLysD competent cells (New England Biolabs, MA, USA). Protein expression was induced at OD = 0.4 with 0.2 mM IPTG for 4 h at 37 °C. Bacterial cells were then pelleted and sonicated in lysis buffer containing 400 mM NaCl, 50 mM Tris pH 7.2, 1% Triton X-100,1 mM EDTA, 1 mM DTT, 50 μM PMSF, 5 mM benzamidine hydrochloride hydrate, and 3 μM aprotinin. Lysates were centrifuged at 20,000 × *g* for 45 min at 4 °C and the supernatant was further cleared by passing through a 0.45-micron filter. The filtered supernatant was incubated overnight at 4 °C with glutathione affinity matrix. The matrix was washed with buffer containing 50 mM Tris pH 7.6, 50 mM NaCl, and 5 mM MgCl_2_, and the GST-tagged fusion protein was eluted from the matrix by incubation with 30 mM reduced glutathione.

Recombinant GST-Mitf and His-CENP-A (Sigma) interaction was performed as described before^25^. Briefly, equal molars of GST-Mitf or GST-EV and His-CENP-A were incubated in 500 µl reaction buffer containing 20 mM Hepes (pH 8.0), 150 mM NaCl, 2 mM MgCl_2_, 1 mM DTT, 50 μM PMSF, 5 mM benzamidine hydrochloride, 3 μM aprotinin, and 1% Triton X-100 overnight at 4 °C. After the incubation, prewashed glutathione agarose beads were added, and further incubated for 4 h at 4 °C. The GST beads were washed extensively with reaction buffer and the proteins were eluted with SDS–PAGE sample buffer and analyzed on 10% SDS–PAGE.

### Gel filtration chromatography

The gel filtration experiment was performed, as described previously^32^. Gel Filtration Calibration Kit (GE Lifesciences Cat. No. 28-4038-42) was used to detect the retention times on Coomassie-stained SDS–PAGE protein gels.

### Statistical analysis

All quantitative data were presented as the mean ± SEM of at least three independent experiments by Student’s *t* test for between group differences and analysis of variance for comparisons among three or more groups. The *p* < 0.05 was considered as statistically significant.

## Data Availability

The datasets for the current study are available from the corresponding author upon reasonable request.
